# AntiJen: a quantitative immunology database integrating functional, thermodynamic, kinetic, biophysical, and cellular data

**DOI:** 10.1186/1745-7580-1-4

**Published:** 2005-10-06

**Authors:** Christopher P Toseland, Debra J Clayton, Helen McSparron, Shelley L Hemsley, Martin J Blythe, Kelly Paine, Irini A Doytchinova, Pingping Guan, Channa K Hattotuwagama, Darren R Flower

**Affiliations:** 1 Edward Jenner Institute for Vaccine Research, High Street, Compton, Berkshire, RG20 7NN, UK

**Keywords:** B Cell Epitopes, MHC-peptide binding, T Cell Epitopes, MHC, TCR, Antibodies, Vaccines

## Abstract

AntiJen is a database system focused on the integration of kinetic, thermodynamic, functional, and cellular data within the context of immunology and vaccinology. Compared to its progenitor JenPep, the interface has been completely rewritten and redesigned and now offers a wider variety of search methods, including a nucleotide and a peptide BLAST search. In terms of data archived, AntiJen has a richer and more complete breadth, depth, and scope, and this has seen the database increase to over 31,000 entries. AntiJen provides the most complete and up-to-date dataset of its kind. While AntiJen *v2.0 *retains a focus on both T cell and B cell epitopes, its greatest novelty is the archiving of continuous quantitative data on a variety of immunological molecular interactions. This includes thermodynamic and kinetic measures of peptide binding to TAP and the Major Histocompatibility Complex (MHC), peptide-MHC complexes binding to T cell receptors, antibodies binding to protein antigens and general immunological protein-protein interactions. The database also contains quantitative specificity data from position-specific peptide libraries and biophysical data, in the form of diffusion co-efficients and cell surface copy numbers, on MHCs and other immunological molecules. The uses of AntiJen include the design of vaccines and diagnostics, such as tetramers, and other laboratory reagents, as well as helping parameterize the bioinformatic or mathematical *in silico *modeling of the immune system. The database is accessible from the URL: .

## Introduction

There is a vast, and ever increasing, volume of important information that has accumulated from decades of experimental analysis within immunology. This will only become compounded as high-throughput techniques begin to impinge upon the immunological biosciences. The only efficient way for this information to be properly utilized requires the development of databases that store it and systems that use it. Although the type of data archived may alter from case to case, nonetheless the creation, use, and manipulation of databases containing biologically important information is the most crucial feature of current bioinformatics, both as it supports the genomic and post-genomic revolutions and as a discipline in its own right. There is nothing new in developing databases focusing on immunology: many spotlighting the in-depth sequence analysis of individual immunomacromolecules have existed for some time [1]. Functional or epitope-orientated databases are a more recent development. Examples include the now defunct MHCPEP database [2], FIMM [3], SYFPEITHI [4], the HIV sequence database [5], the HLA ligand database [6], the EPIMHC database [7], and the MHCBN database [8].

An epitope is any molecular structure that can be recognised by the immune, or other biological, system. Epitopes, or the antigen from which they are derived, can be composed of protein, carbohydrate, lipid, nucleotide, or a combination thereof. It is through recognition of foreign, or non-self, epitopes that the immune system can identify and, hopefully, destroy pathogens. Hitherto, peptide epitopes have been the best studied, and have, traditionally, have been categorized as either T cell or B cell epitopes. T cell epitopes are peptides presented to the cellular arm of the immune system via the MHC-peptide-TCR complex. B cell epitopes represent surface regions of an antigen that are bound by soluble or membrane-bound antibodies. If this region of a protein antigen is comprised of residues distally separated within the primary structure, and brought into local proximity by protein folding, then it is termed a discontinuous or conformational B cell epitope. Linear or continuous B cell epitope residues are sequential in both primary structure and thus as a region on the proteins' surface. Such epitopes are predominantly identified by antigen-specific antibody cross-reactivity with peptides.

There is a need to create a databank for the wider disciplines of immuno-vaccinologists, which can act as a central repository and resource. Our aim is to complement other databanks [2-8] and thus we have developed AntiJen, a computational information resource for immunology and vaccinology that integrates quantitative kinetic, thermodynamic and biophysical data, with functional and cellular information. AntiJen *v2.0*, a development of our earlier database system JenPep [9,10], contains functional data on T cell and B cell epitopes. Moreover, the B cell archive is now sub-divided into linear and conformational epitopes. These epitopes form the basis of the humoral immune response and, unlike T cell epitopes, methods of prediction are often inaccurate [11]. A more in-depth B cell epitope archive should aid the development of prediction strategies. Antigen recognition by the Major Histocompatibility Complex (MHC) is vital to T cell activation hence, the inclusion of thermodynamic data on the binding of peptides to MHC molecules and T Cell Receptor (TCR) binding to peptide-MHC (pMHC) complexes. This data is complemented by kinetic data based on the same molecular interactions. Data on antigen processing and presentation is also included in AntiJen. Binding data derived from peptide interactions with the Transporter Associated with Antigen Processing (TAP transporter) are included in the archive. Additionally, quantitative specificity data from position-specific peptide libraries is included. AntiJen also incorporates thermodynamic data on protein-protein interactions, within an immunological context, such as co-receptor and superantigen binding, plus interactions with the MHC. All of these interactions are, potentially, key factors for the successful computational design of vaccines.

AntiJen also contains biophysical data, including diffusion coefficients and cell surface copy numbers, on a variety of immunological molecules. Such data provides insight into the number of target receptors, which is an important, if under explored, component of binding between cells. Indeed, the number of molecules expressed on the membrane can alter depending on disease. The final addition to the databank focuses upon antigen binding to antibodies. One key innovation is a greatly increased compendium of experimental conditions, which, in conjunction with a greatly enhanced search capacity, consolidates our databases as a unique, value-added data source, fostering developments within both *in silico *immunology and the wider community of immunovaccinology. The database is available from the URL: .

## Database development

Relative to the database system used for JenPep [9,10], the interface to AntiJen is entirely new, having been completely rewritten. AntiJen has been designed and implemented using postgreSQL, a system comprising a relational database and database server, and has thus established increased database robustness, creating an improved infrastructure for foreseeable issues of data storage and data growth. Data within AntiJen is structured into twenty-four normalised tables. Each is category specific and holds either statistical or experimental data. Additional tables accommodate the keyword data – which powers our protein-orientated antigen search and allows integration of the BLAST search – and there is also a structural data table to accommodate links to external structural databases. The user interface consists of a series of HTML forms. The search requests from these forms target PERL scripts integrated with SQL which in turn query the database.

## Database content

Compared to its progenitor JenPep [9,10], the data archived in AntiJen *v2.0 *has grown considerably in depth (additional data types such as experimental conditions), breadth (addition of new data to existing databases), and scope (addition of extra sub-databases containing novel kinds of information). Additions to AntiJen have been derived from exhaustive searching of the primary literature, to give a dataset of > 31,000 entries. AntiJen *v*2.0 now consists of 11 sub-databases; details of the different databases are given in Table 1. The relative sizes of the databases and the growth from JenPep are summarised in Table 2.

**Table 1:** AntiJen sub-databases and content.

**DATABASE**	**CONTENT**
T Cell Epitopes	Contains T Cell epitope peptides (known binders).
B Cell Epitopes	Contains B Cell linear and conformational epitope peptides.
MHC-Peptide	Binding data relating to antigenic peptides and MHC interactions.
TCR	Binding data relating to antigenic peptides – TCR – MHC interactions.
TAP	Binding data relating to antigenic peptides and TAP interactions.
Kinetics	Kinetic binding data for MHC peptide interactions.
IPPI	Binding data for a collection of immunological protein interactions.
Diffusion Coefficient	Collection of Diffusion and Friction coefficients for surface peptides.
Copy Number	Number/Abundance of cell surface molecules.
Peptide Libraries	Relative binding data for antigenic peptide amino acid substitutions.
Antibody-Peptide	A variety of antibodies known to bind proteins.

**Table 2:** Size of AntiJen relative to JenPep. The number of peptides for each category in the AntiJen database is given, distinguishing between class I and class II categories, where appropriate. Growth versus JenPep 1 and 2, the progenitors of AntiJen, is included. For certain data categories, most obviously TAP binding data, re-evaluation of the quality of data within JenPep has seen it decrease rather than increase, however the expansion of the data is clearly seen.

**DATABASE**	**JenPep *v1.0***			**JenPep *v2.0***			**AntiJen *v1.0***			**AntiJen *v2.0***		
	**Class 1**	**Class 2**	**Total**	**Class 1**	**Class 2**	**Total**	**Class 1**	**Class 2**	**TOTAL**	**Class 1**	**Class 2**	**TOTAL**
**T cell epitotes**	1266	795	2061	2060	1158	3218	2247	1578	3825	2402	1585	4158
**MHC peptide binding**	3196	2652	5848	6411	5925	12336	6853	7772	14625	7304	8114	15454
**TAP peptide binding**			432			441			408			1106
**B cell epitotes**						816			1295			3541
**TCR – peptide-MHC**						49	375	124	594	527	253	782
**MHC peptide kinetics**							704	243	947	897	294	1150
**IPPI**									805			2675
**Copy Number**										161	243	414
**Diffusion coefficients**												759
**Peptide Libraries**												897
**Antibody**												395

AntiJen contains both generic and dataset-specific data. For each entry, we record the peptide sequence (eg. *YTSDYFISY*) of the epitope using the standard one-letter code, its length (9 in this case), and, by linking to the sequence database Swiss-Prot  or NCBI , the antigen to which the peptide sequence most closely matches (in the case of *YTSDYFISY*: C-ests-1 (p54), *SWISS-PROT code *P41156). The description of the antigen is, wherever possible, obtained directly from the literature. AntiJen is also linked to PUBMED. This allows us to record the original citation associated with the data. For example, for *YTSDYFISY*, we cite: *Journal of Immunol 1994 volume 152 pages 3913–3924, PUBMED ID 8144960*. For the T cell epitope, MHC ligand, and TCR-pMHC complex categories, we also record, for each peptide, the MHC restriction in terms of the host species, class (class I vs. class II), and, where the data is available, the serotype and allele. For *YTSDYFISY*, these data would be *human*, *class I*, HLA-A1, and A*0101.

Entries within AntiJen are, in turn, linked to external databases, which enables further in-depth cross referencing. As we have said, protein sequence identifiers, which may be the source of an antigenic peptide or immunological co-receptor, link directly to details in the Swiss-Prot database [12] or the NCBI protein database. Journal references can be viewed via a link to the PUBMED database , and thus to full literature references, where available. AntiJen also links to structural data, currently derived from the MPID database [13] and the Protein Data Bank [14]. The database aims to provide access to background data where available.

Allele identifiers serve as a link to the IMGT/HLA database  at the European Bioinformatics Institute [15]. Inherent variability in the way MHC alleles are named within the primary literature  prevents us from unambiguously standardizing nomenclature within AntiJen. HLA nomenclature follows that of the HLA Informatics Group . An allele is named using a defined pattern. For example, for HLA-A*0101: the HLA-A refers to the HLA locus; the first 01 to the serologically recognized A1 antigen and the final 01 to the individual HLA allele protein sequence. AntiJen stores the antigen classification (i.e. HLA-A1) and, when available, the specific allele. We have often encountered problems with the nonstandard allele reporting. A 4-digit HLA name necessarily implies the two digit serological antigen, a two digit classification clearly does not imply a specific allele.

During database compilation, a sequence search allows us to identify the protein from which an epitope sequence originates. However, because epitopes are generally short, their sequences may be present in several potential antigens: in orthologues, paralogues, or in totally unrelated sequences. As epitopes are processed from whole proteins via a complex proteolytic pathway, one can use the sequence context to infer preferred proteasomal or endoplasmic protease cleavage patterns, but not if its context is defined incorrectly. Moreover, assuming that AntiJen is used subsequently to assign the antigenic status of proteins, wrongly identifying particular proteins as antigens can lead to the percolation of annotation errors [16,17].

AntiJen is, where possible, a quantitative database archiving continuous measures of binding. This is a fundamental feature of several sub databases, such as the MHC ligand and pMHC-TCR databases. The binding of an immunological macromolecule to a peptide or other biomacromolecule is quantified as are other receptor-ligand interactions:

*R*+*L*↔*RL*

Here R is the receptor (an MHC or TCR), L the ligand (peptide or pMHC), and RL, the receptor-ligand complex (pMHC or pMHC-TCR complex). The rate of the forward reaction is proportional to [L] [R]. The rate of the reverse reaction is proportional to [RL] as no other species are involved in dissociation. At equilibrium, the forward and reverse rates are equal, and so using k_on _and k_off _as the respective constants:

*k*_*on*_[*R*][*L*] = *k*_*off*_[*RL*]

Rearranging:



Here K_D _is the equilibrium dissociation constant, which represents the concentration of ligand that occupies 50% of the equilibrium receptor population, and K_A _is the equivalent association constant.

Experimentally, the measurement of equilibrium dissociation constants is often addressed using radio-ligand binding assays. Saturation radio-ligand binding assays measure equilibrium binding, at a range of peptide concentrations, to establish affinity (K_a_) and receptor number (B_max_). Competitive binding experiments determine binding at a single labelled ligand concentration in the presence of a range of concentrations of unlabelled ligand. AntiJen records a hierarchy of different binding measures in its different sub-databases. Equilibrium constants are the most dependable and sit atop this hierarchy. Next come IC_50 _values, which can be obtained from a competitive radio-ligand or fluorescence assay. These are the Fmost commonly reported binding measures.

Values obtained from radio-ligand or fluorescence methods may be significantly different. IC_50 _values for a peptide may vary between experiments depending on the intrinsic affinity and concentration of the standard radiolabelled reference peptide, as well as the intrinsic affinity of the test peptide. IC_50 _values vary with the equilibrium dissociation constant, at least within a single experiment. In practice, the variation in IC_50 _is often small enough that values can be compared between experiments. For the peptide discussed above, *YTSDYFISY*, the radiolabelled IC_50 _value recorded in AntiJen is 5.3 nM. BL_50 _values are also obtained from a peptide binding assay and are commonly reported. They are the half maximal binding levels calculated from mean fluorescence intensities of peptides binding to MHCs bound on the surface of RMA-S or T2 cells. Cells, pre-incubated with peptides, are labelled with a fluorescent monoclonal antibody. An overview of the thermodynamic and kinetic binding data within AntiJen is given in Table 3.

**Table 3:** AntiJen Thermodynamic and Kinetic Data. An overview of the 6 AntiJen databases that provide binding data. It must also be noted that several of the databases contain additional data not present in any of the other databanks.

	**MHC-Peptide**	**Kinetics**	**IPPI**	**TAP**	**pMHC-TCR**	**Antibody**	**TOTAL**
**IC_50_**	8562	0	247	1000	0	4	9813
**K_on_**	0	188	563	0	157	87	995
**K_off_**	0	146	610	0	150	101	1007
**K_D_**	359	156	1143	16	227	70	1971
**K_a_**	65	0	37	0	28	132	262
**t_1/2_**	0	207	72	0	148	0	427

**Figure 1:**
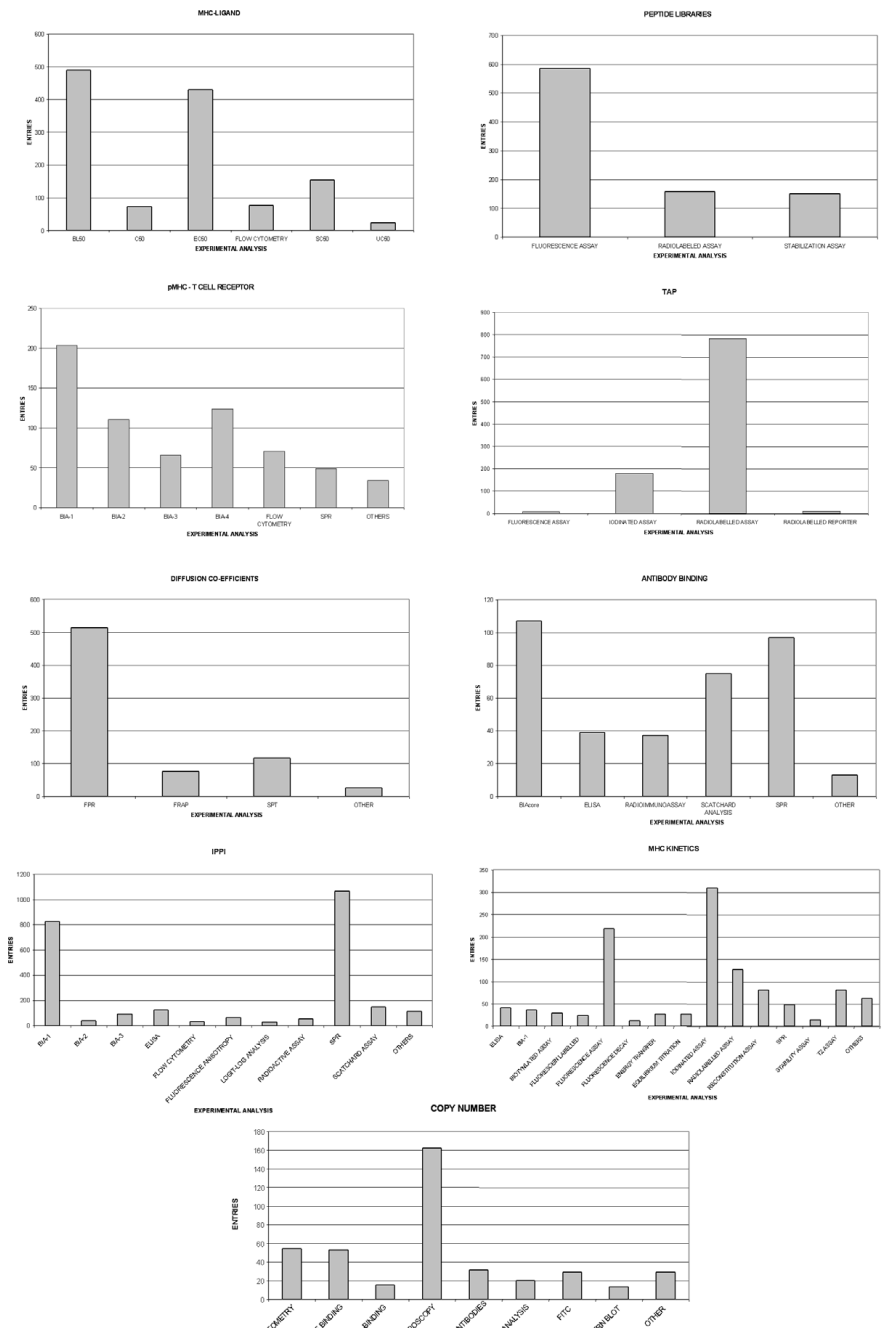
**The distribution of experimental methods applied within each database**. The number of different experimental methods and the abundance of data relating to the method is shown within the figures. The '*OTHERS*' category refers to methods for which there is a relatively small number of entries.

AntiJen also now contains experimental conditions, such as temperature and pH. A summary of this data is given in Table 4. The accuracy of data depends greatly upon the experimental method used. The grouping of data with respect to specific experimental techniques allows a more thorough assessment of training sets. Figure 1 shows the distribution of data for each type of analysis with respect to each database. The MHC Kinetics and TAP databases highlight the problems outlined above. The kinetics database contains data determined from over 14 methods while the TAP database is derived from 4 methods, with radiolabelled assays accounting for 80% of the data.

**Table 4:** Experimental conditions and associated information archived in AntiJen. Number of recorded experimental conditions stored within the AntiJen database. For each condition (temperature, pH, *etc*.) we show here the number of entries within a particular sub-database. [Standard] is the concentration of labelled standard peptide in an assay. Likewise, [competitor] is the concentration of competitor peptide within a competition assay. [peptide] is the concentration of peptide in a kinetic experiment. The Method category refers to a standard procedure used to perform a particular assay. Differences in the number of recorded data, relative to figures in Table 1, arise primarily from the omission of key details from particular papers. Archiving of experimental conditions is on-going.

**DATABASE**	**TOTAL**	**pH**	**Temperature**	**[standard]**	**Stand. peptide seq.**	**[competitor]**	**Method**	**[peptide]**
**MHC Binding**	15454	6679	9831	10893	12796	5007	1251	
**MHC Kinetics**	1150	677	1101				1149	606
**TAP Binding**	1106	22	243	1092	1101	86	981	
**TCR-pMHC**	782	426	632				668	
**IPPI**^*a*^	2675	726	1371				2600	
**Copy Number**	414	183	278				414	
**Peptide Libraries**	897		897				897	
**Diffusion Coefficient**	759	321	668				736	
**Antibody**	395	119	115				372	

The compilation process has highlighted the considerable inconsistency within the immunological literature regarding the recording of such fundamental data. AntiJen contains, however, a direct, verbatim transcription of data from the primary literature. As such, we do not attempt, as a matter of policy, the comprehensive and retrospective correction of potential errors. To undertake such correction would only compound any errors, introducing the kind of percolating inconsistencies so much a feature of other database systems [16,17]. Further inaccuracies may stem from our logistic inability to verify data independently, therefore we must trust those values reported in the literature.

### Subsidiary Databases in AntiJen

The AntiJen database contains a number of sub-databases. Each of these contains data on different aspects of the biological function and/or biophysical properties of different classes of immunomacromolecule. We describe the nature and content of each sub database below.

### B Cell Epitopes

Epitopes are the principal chemical moieties recognized by the immune system. Although the importance of non-peptide epitopes, such as carbohydrates and lipids, is now increasingly well understood, peptidic B cell and T cell epitopes remain the principal tools by which the intricacy of the immune response can be explored. B cell epitopes are regions of the surface of a protein, or other biomacromolecule, recognized by soluble or membrane-bound Antibody molecules. In developing AntiJen, we have discarded the contents of our previous B cell archive and constructed one *de novo*. It contains an entirely new data set with a substantially different data structure. There are two forms of epitopes: linear and discontinuous. A linear B cell epitope is composed of a single stretch of sequential residues. A discontinuous B cell epitope is composed of sequentially separate residues brought into close proximity in a conformationally-dependent arrangement. The data we archive is primarily focused upon linear epitopes. This is due to the far greater amount of experimental data available for linear epitopes, which reflects both the relatively facile experiments needed to identify them and an implicit belief in their utility as potential vaccine epitopes. By contrast, discontinuous epitopes are thought to be more prevalent within folded proteins, but are far more challenging to determine experimentally. The archive catalogues the sequence of binding peptides, and also gives the length and source: *TTGDVIASS*, a 9 amino acid peptide from *Escherichia coli *non-fimbrial adhesion. Residues identified as important in binding to the antibody are recorded. This may correspond to a whole peptide or a subsequence, such as *TTGDVI *in the above example. The peptides are also categorized in terms of their relative observed immunodominance. Antibodies host organism and isotype are recorded. The current B cell epitope archive contains 3,541 epitopes.

### T Cell Epitopes

T cell epitopes are short peptides bound by major histocompatibility complexes (MHC) and subsequently recognized by T cells. Epitopes recognized by both CD4+ and CD8+ T cells are included in the database. Such epitopes can be identified in many different ways. However, this diversity of measurement imposes a certain need for consistency, necessitating the requirement for recording a range of different experimental methods. The archive has expanded to include 4,158 entries. The entries contain the epitopes, ranging in length from 4 to 38 amino acids, peptide information, detailing the source, with links to Swiss-Prot and the corresponding MHC restriction data such as Serotype, Allele and Class. Additionally, the peptides are categorized in to groups such as Allergens, Bacterial, Cancer, Human, Viral and Self peptides.

### MHC – Peptide binding

AntiJen continues to archive quantitative data on the thermodynamics of peptide interactions [18,19], and it has expanded in number and content, with additions such as experimental conditions, plus specific Standard and Competitor peptide concentrations used in the assays. The current archive contains 15,454 entries. The sequence of the binding peptide, along with the source, plus relevant MHC restriction data is recorded. The restriction alleles currently include those from Human, Mouse, Rat, Rhesus Monkey, Cotton-top Tamarin, and Chimpanzee. AntiJen contains IC_50 _values, binding affinity measurements from competitive binding assays, for which the standard and competitor peptides and concentrations are recorded, plus BL_50 _values, calculated from peptide stabilizing assays. Where possible, antibodies and the concentrations used to calculate BL_50 _values are archived. Additionally, but on a somewhat smaller scale, equilibrium association (K_A_) and dissociation (K_D_) constants are recorded for peptide-MHC interaction. Melting temperatures (T_m_) and signal wavelength are also recorded; this is the temperature and wavelength at which 50% of the MHC protein is denatured as measured by circular dichroism. AntiJen also records so-called Weak/Non-binders. This indicates that the peptide has been tested in an MHC restriction assay and has been found to exhibit a binding affinity, i.e. an IC_50 _value > 10,000 nM for a radio-ligand assay, so low that it can be categorized as inactive.

### pMHC-T Cell Receptor interaction

The TCR sub-database contains 782 entries, which records thermodynamic and kinetic binding data for the interaction of peptide-MHC (pMHC) complexes with TCRs. Different MHCs exhibit a distinct selectivity for certain peptide sequences. T cell receptors, in their turn, also exhibit different affinities for peptide-MHC complexes. The entries contain epitope, peptide source and MHC restriction data, as described above, plus TCR structure information, located at the MMBD database . Furthermore, any mutations are noted and a designated name for the TCR is archived. In addition, the peptides are recorded as either agonists or antagonists. The binding data is given as equilibrium constants (K_D_), EC_50 _values, rate of association (K_on_), rate of dissociation (K_off_), association constant (K_A_) and the half-life (t_1/2_) of the TCR-peptide interaction.

### TAP Binding

This dataset contains binding data for the interactions between peptides and the TAP transporter, one of the principle steps in antigen presentation. As with the peptide-MHC database, the data is established from competitor binding experiments, based on labelled assays. Therefore, standard and competitor peptide sequences and their concentrations are recorded. The binding data is given as IC_50 _and K_D _values. The database currently contains 1,106 entries, with peptides from Human, Rat and Mouse sources. Based on IC_50 _values > 10,000 nM, the peptides are categorized as weak/non-binders. The entries have increase in number from the level found in JenPep, although several entries were removed in an effort to increase the accuracy and consistency of the archive (Table 2).

### Peptide-MHC Kinetics

AntiJen's kinetics sub-database, which contains 1,150 entries, mostly relates to Class I MHC data. It records measurements for forward and reverse rate constants for complexation events. This complements the thermodynamic measurements on peptide-MHC binding described above. The data currently focuses upon both the half-lives of binding interactions, as well as association and dissociation rate constant values (K_on _and K_off_) for the recorded epitopes. Additionally, concentrations of the peptide, MHC and TAP are archived. The half-life for radioisotope labelled β_2_-microglobulin dissociation from an MHC class I complex, as measured at 37°C, is also archived. This is a kinetic measurement rather than a thermodynamic one, although it is often assumed that the greater the half-life the stronger the peptide-MHC complex. The half-life (t_1/2_) equals:



Here the t_1/2 _corresponds to the dissociation of the MHC-β_2 _microglobulin complex rather than the kinetics of the protein-ligand interaction, but is still peptide dependent, as well as kinetic in nature.

### Immunological Protein-Protein Interactions

The immune system is built around protein interactions therefore we developed another new sub-database which deals with Immunological Protein-Protein Interactions (IPPI). This archive contains 2,675 entries based on a variety of binding data, such as K_on _and K_off _rates, for a range of macromolecules implicated in physiological or pathological interactions, as well as K_D_, K_A _and IC_50 _values. The molecules include receptors such as CD4 or CD8 molecules, superantigens and other microbial virulence factors, cytokine receptors and cell adhesion molecules. The entries detail both protein partners involved in the binding interaction, with links provided to the NCBI-Entrez database. Additional data for MHC receptors is archived, whereby the reactive epitope is recorded and the co-receptors are categorized into viral, bacterial and self peptides. MHC data outlined in the previous databases is given, where appropriate including any mutations to the MHC.

### Antibody – Protein Binding

AntiJen also contains a further sub-database, which comprises thermodynamic data relating to antibody-antigen binding. The dataset contains 395 entries for antigen proteins and antibodies, mostly derived from viral and mammalian sources. Reported values were obtained using radiolabelled assays and BIAcore analysis. This archive should aid in the selection of antibodies and peptides for *in vitro *studies. The entries list the antibodies and the binding/kinetic data, consisting of K_D _and K_A _values and to a lesser extent K_on_, K_off _and IC_50 _values.

### Peptide Libraries

This archive further complements our MHC binding databases by indicating the relative contribution of residues within peptide libraries to MHC binding. 897 entries contain quantitative specificity data derived from position specific peptide libraries [20]. This catalogues the relative effect on affinity, in the form of IC_50_, log relative SD_50 _and log SI values, of all substitutions, at all peptides positions, against a random sequence backdrop. All of the libraries relating to a known peptide binder are designated a name within AntiJen, this usually consists of the author and year of publication. The archive contains the core peptide, along with the mutation position and the substituted amino acid. The corresponding MHC data is given as mentioned above.

### Diffusion Co-efficients

To further increase the range of data archived, AntiJen also contains 759 records of cellular biophysical data, in the form of diffusion co-efficients, recorded as cm^2^s^-1^, for a diversity of cell surface molecules, including MHCs (Mouse and Human), viral peptides and other receptors [21]. The molecules are either chemically or fluorescently labelled and then measured using one of two methods: Single Particle Tracking (SPT) or Fluorescence Photobleaching Recovery (FPR). SPT monitors the lateral motion of a labelled molecule while FPR measures the rate of subsequent infiltration from a photobleached section of the membrane. Friction co-efficient data is also given, which measures of the velocity and force applied to an antibody-coated bead. Records contain the cell or cell type where diffusion is occurring, the name of the diffusing protein along with the form of labelling applied. Furthermore, specific experimental data is given such as antibody bead size. In this case the diffusion of the beads is monitored. Increasingly, data relating to photobleaching is included, such as beam power, bleaching duration, pre- and post-bleach time, *etc*..

### Copy Numbers

The final sub-database contains 414 measures of cell surface populations of different molecules, called cell surface copy numbers hereafter. This database focuses on an array of molecules, including Class I MHCs (22) and TCRs [23]. The entries are given as number and type of MHC molecules, number of MHC-peptide complexes or abundance of peptides associated with each MHC serotype, generally defined by mass spectroscopy. The entries list the cell type, the antibody bound to the MHC and, if appropriate, the binding epitope. This only applies to the number of MHC-peptide complexes and the abundance of peptides associated with each MHC.

## Searching the database

Search mechanisms within AntiJen are significantly improved and allow either a detailed or a broad search from a simple user interface. From our experience with JenPep, we recognize that accessibility to the data in a user friendly manner is a vital requirement, and have improved our current search mechanisms and developed new search interfaces. Two different search mechanisms are available. One is based on BLAST [24] and the other is a bespoke system, allowing several alternative searches. Within a typical search, the user-entered search criteria are carried from an HTML form to a category specific PERL/SQL script, which performs the database queries.

**Figure 2:**
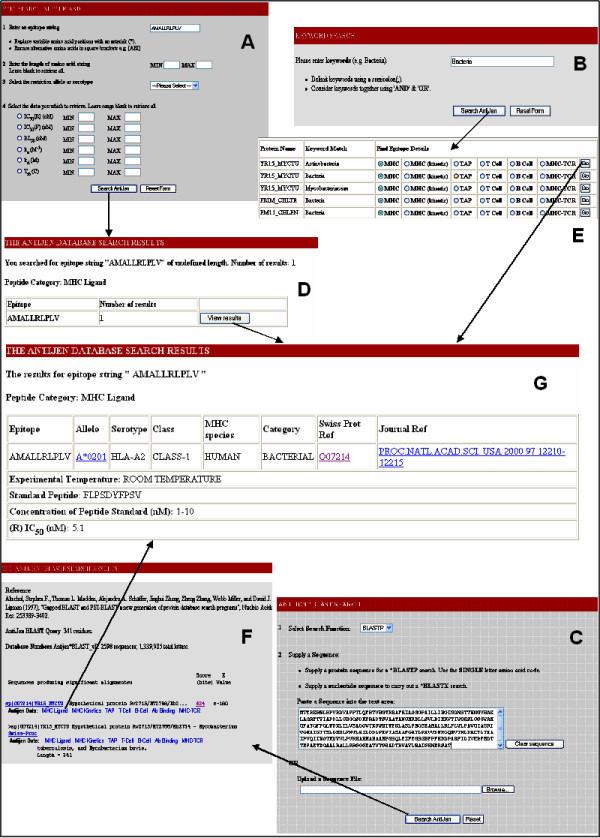
**Overview of the different search methods within AntiJen**. The example search is focused upon an MHC ligand. The MHC ligand data can be searched directly (A) from a link on the AntiJen homepage, a broad search and specific search is available. A search for the epitope AMALLRLPLV, has one hit (D), this leads to the entry (G). All of the other sub-databases can be searched in this manner from the homepage. The two other searches are more generalised. A Keyword search (B) carries out a broad search on the whole database, for the criteria – Bacteria. This search gives 139 hits (E) and all of the sub-database entries can be selected from this output. The final search method is a BLAST search (C). The peptide (or nucleotide) sequence is queried against a local protein database. The output (F) provides links to the sub-databases relative to the protein.

The BLAST search allows querying of a peptide or nucleotide sequence against the proteins contained in AntiJen; all entries containing data within AntiJen which are relevant to a protein sequence are linked via the BLAST output. A local database of protein sequences is searched with BLASTP or BLASTX [24] using the BLOSUM64 matrix. All BLAST control variables are fixed. An HTML Front-End (Figure 2), where a sequence can be entered or uploaded, connects to a web server-based PERL/CGI scripts, which interacts with BLAST. An annotated version of the default BLAST output is produced and links to AntiJen entries via SWISS-PROT [12] accession codes, which act as a query within a Keyword search. This allows AntiJen entries to be viewed directly from the BLAST output.

**Figure 3:**
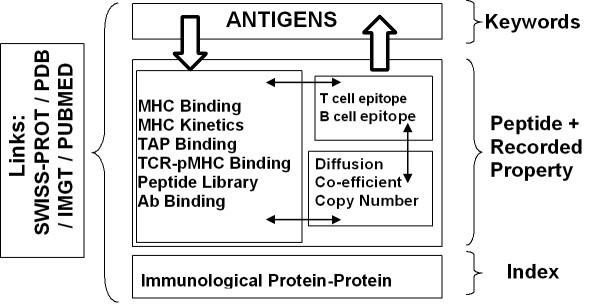
**Searchable database types within AntiJen**. The database contains 3 types of searchable sub database: a set of Antigens searchable by keyword, various databases of functional and thermodynamic data searchable by peptide sequence, and a database of immunological protein-protein interactions searchable through an index. Peptide sequence searches can be explicit or "motif" based. Searches can also be focussed by setting the value ranges for properties, such as IC_50 _etc, recorded in the databases. Currently there is a link between all the thermodynamic and kinetic databases and between them and the database of antigens. Only antigens with data in one of the other sub-databases are included in the antigen database. Links to external databases are also indicated. The BLAST search provides an overall search of the databases except the Protein-Protein Interactions archive.

**Figure 4:**
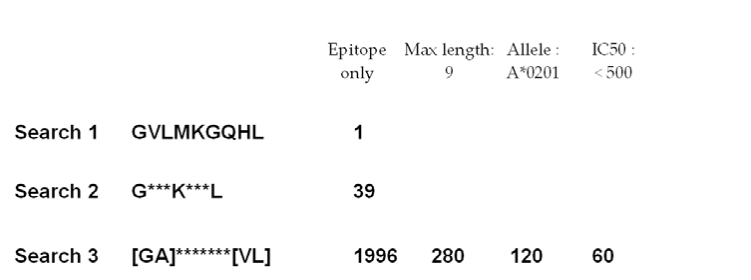
**Sparse peptide sequence search**. Examples of the two search types available in AntiJen: (Search 1) a substring query and (Searches 2 and 3) PROSITE-like sparse queries allowing sets of variable (indicated by asterisks) and alternative (encased in square brackets) amino acids in the search. In our example, each query is an extension of the previous search. The initial search type returns a single hit, which is a weak binder. By introducing variable amino acid positions within the query string, the second query permits access to a larger data set with 39 hits being returned. The third search utilises both variable and alternative amino acid request functionality and returns 1996 hits. The number of entries returned can be reduced by specifying the epitope length, limiting IC_50 _values and restricting by one MHC allele. This search is constrained to peptides of amino acid length 9, which returns 280 hits. Constraining further by MHC allele HLA-A*0201, reduces this to 120. An additional constraint using the IC50 data filter, and requesting values below 500 only (the epitope range), reduces this again to 60.

At present, peptide string, keyword, and protein name index searches are available within the bespoke system, which allows querying of individual peptide sequences or at the level of whole protein antigens. An overview of the AntiJen search systems is given in Figure 2 and 3. Epitopes, MHC, TCR, TAP peptide binding and kinetics databases can all be searched using sequence strings. The search protocol first returns an epitope list and a count of epitope matches. Subsequently, experimental criteria can be accessed for each selected epitope. Peptides can be searched using an amino acid orientated query: a sparse peptide string, similar in form to a peptide binding motif [3] or a PROSITE pattern [25], is used to identify all matching sequences. See Figure 4. Alternately, a list of protein antigens within AntiJen can be searched using keywords; the thermodynamic binding data such as MHC-peptide, TCR-pMHC and TAP, plus the B and T cell archives, related to the search criteria can then be selected from the matches based upon the SWISS-PROT accession codes [12], displaying all corresponding entries in the database.The other search method, allows the IPPI, diffusion co-efficients, peptide libraries, antibody-protein and copy number sub-databases to be searched using an index method, within a user-friendly HTML drop-down menu. Each of these methods can also be moderated using subsidiary search filters, data size ranges, and result presentation alternatives, such as peptide length or IC_50 _values. Minimum and maximum values can be used to restrict results, as can selection of MHC restriction alleles.

## Discussion

The name given to our new database, AntiJen, reflects a shift from a peptide orientated database structure, which was inherent within our earlier JenPep database, to one which can properly balance its focus on both protein antigens and isolated peptides. As such, it represents an important, integrated, immunological data resource. AntiJen now provides broad insight into both T cell and B cell mediated antigen recognition. In addition, through the auspices of the IPPI sub-database, the database also throws light on co-stimulation by co-receptors and gives important insights into the innate immune response. Our approach to protein-protein interactions, focusing on measured affinities, complements other methods, such as the Yeast-2-Hybrid system, which, while giving greater volumes of data, has problems of accuracy [26]. This, while not devoid of experimental artifact, gives a usefully different perspective on cataloguing protein-protein interactions. The further addition of weak binding notation to the MHC-peptide and TAP provides a greater overview of the nature of antigenic epitopes. This is further improved by the addition of the peptide libraries database, whereby key peptide residues can be highlighted. New databases have expanded the breadth of AntiJen to include biophysical data such as diffusion co-efficients and cellular data such as abundance of molecules. The antibody-antigenic protein sub-database will also provide a key resource for *in vivo *and *in vitro *studies, aiding in the selection of antibodies and peptide/protein targets.

AntiJen distinguishes itself from the other specific binding databases [2-8] in several ways. Firstly, more data is recorded; our MHC-peptide database contains over 2,000 more entries than MHCPEP [2] and 10,000 more entries than EPIMHC [7]. Additionally, we have not restricted our archive to only high binders or to a specific category, as seen in EPIMHC and the HIV sequence databases [5]. Furthermore, AntiJen is currently a curated database, which is constantly expanding.

Most obviously, AntiJen is useful in the design of epitope and subunit vaccines. Additionally, AntiJen is helpful in the design of clinical diagnostics and other laboratory reagents, such as the selection of peptides for tetramer design. AntiJen is also useful in the parameterization of mathematical models in theoretical immunology [27]. The redevelopment of the database has focused not only on content, but also on infrastructure. The current system, based on epitope string, keyword and index searches, along with an overall BLAST search, plus the redesigned HTML interface, leads to much greater accessibility and usability. Finally, the database acts as a repository of quantitative, continuous data, for the development of data-driven *in silico *predictive models, such as prediction of epitopes and MHC binding [18,19,28,29] through QSAR modeling.

## Future work

Future tasks in the development of AntiJen, fall into two principle categories: eliminating deficiencies, errors, and inconsistencies within the database and simultaneously reinforcing it by expanding its depth, breadth, and scope. We also need to monitor updates within external databases, so that any alterations are mirrored within the archive. Like all other such repositories, AntiJen is prone to both systematic and random errors within the data accumulation process. User feedback and our interactions with immunologists will hopefully address persisting errors. Deficiencies in our database include our current inability to encode chemically or post-translationally modified peptides, non-natural MHC mutants and non-amino acid peptidomimetic MHC ligands. Additionally, it would also be interesting to complement our existing data on TAP binding with information on antigen presentation pathways, such as proteasomal and cathepsin cleavage patterns. Moreover, the compilation of B cell or antibody epitope data is an area ripe for robust development. Linear and conformational B cell epitopes are very much larger in number than our current compilation, leaving us scope to greatly increase recorded epitopes.

## Conclusion

The development of a database is always a work in progress. Not simply because the easily accessible literature is typically always increasing, but also because of the desire to capture as much of the existing, but hidden, literature, as possible. In the post-genomic era, the database has formed the bedrock and language of bioinformatics; increasingly databases are coming to underpin our modern understanding of biology as a whole. Traditionally, databases have arisen as a response to need, answering the individual and idiosyncratic questions posed by biologists. However, the history of bioinformatics databases has shown the extraordinarily diverse ways in which archived data can be used.

In creating AntiJen, we were motivated partly by our desire, and the desire of collaborators, to use the data within it to build predictive *in silico *models [16,17,28,29], and partly by a more altruistic desire to generate a useful, integrated database system with a quantitative focus. AntiJen has many potential uses throughout the immunological discipline, from immunoinformaticans to experimental immunologists and vaccinologists. By increasing the degree to which data is machine readable and web accessible, we open up new, and previously unthought-of, avenues for the bioinformatic exploration of immunological data.

AntiJen is a primary data resource, amongst the most complete of its kind, yet, like SWISSPROT [12] or GenBank [30] decades ago, it is still relatively small and offers much scope for improved annotation. We see the database as a foundation from which to consolidate, through time, thus achieving a comprehensive resource of immunological data.
